# Barriers and facilitators of electronic patient-reported outcome measures (e-PROMs) for patients in home palliative cancer care: a qualitative study of healthcare professionals’ perceptions

**DOI:** 10.1186/s12904-023-01234-0

**Published:** 2023-08-04

**Authors:** Letteria Consolo, Stella Colombo, Ilaria Basile, Daniele Rusconi, Tiziana Campa, Augusto Caraceni, Maura Lusignani

**Affiliations:** 1 Department of Biomedicine and Prevention, University of Rome “Tor Vergata”, Rome, Italy; 2 Bachelor School of Nursing, IRCCS, National Cancer Institute, Milan, Italy; 3 Intensive Care Unit, IRCCS, National Cancer Institute, Milan, Italy; 4 High-Complexity Unit of Palliative Care, Pain Therapy and Rehabilitation, IRCCS, National Cancer Institute, Milan, Italy; 5 Urology Unit, IRCCS, National Cancer Institute, Milan, Italy; 6 University of Milan, Milan, Italy; 7 Department of Biomedical Sciences for Health, University of Milan, Milan, Italy

**Keywords:** Palliative care, Home palliative care, Cancer, Electronic patient-reported outcome, End of life, Healthcare professionals

## Abstract

**Background:**

Patient-reported outcomes in palliative care enable early monitoring and management of symptoms that most impact patients’ daily lives; however, there are several barriers to adopting electronic Patient-reported Outcome Measures (e-PROMs) in daily practice. This study explored the experiences of health care professionals (HCPs) regarding potential barriers and facilitators in implementing e-PROMs in palliative cancer care at home.

**Methods:**

This was a qualitative descriptive study. The data were collected from two focus groups structured according to the conceptual framework of Grol. HCPs involved in home palliative cancer care of Fondazione IRCCS Istituto Nazionale dei Tumori of Milan were enrolled. Data were analyzed using a reflexive thematic analysis.

**Results:**

A total of 245 codes were generated, 171 for the first focus group and 74 for the second focus group. The results were subdivided into subthemes according to Grol’s themes: Innovation, Individual professional, Patient, Social context, Organizational context, except Economic Political context. Nine HCPs attended the first focus group, and ten attended the second. According to these participants, e-PROMs could be integrated into clinical practice after adequate training and support of HCPs at all stages of implementation. They identified barriers, especially in the social and organizational contexts, due to the uniqueness of the oncological end-of-life setting and the intangible care interventions, as well as many facilitators for the innovation that these tools bring and for improved communication with the patient and the healthcare team.

**Conclusions:**

e-PROMs are perceived by HCPs as adding value to patient care and their work; however, barriers remain especially related to the fragility of these patients, the adequacy of technological systems, lack of education, and the risk of low humanization of care.

**Supplementary Information:**

The online version contains supplementary material available at 10.1186/s12904-023-01234-0.

## Background

Palliative care (PC) has a holistic view that accounts for various patient dimensions, including physical, mental, social, spiritual, and economic dimensions [1, 2]; it also prevents or treats symptoms and side effects of advanced and chronic diseases [3]. Collection of patient-reported outcomes (PROs) in palliative care can support patient engagement in health care delivery [4–6], providing patients’ perceptions of their health, quality of life, mental well-being, and healthcare experience [6, 7]. Additionally, PROs help healthcare professionals (HCPs), physicians and nurses in decision-making and facilitate communication between the care team and patients/families [6–9].

PRO data can be collected using standardized, validated questionnaires called patient-reported outcome measures (PROMs), defined as direct reports from patients about their health conditions without interpretation by clinicians [2, 5, 10–12]. PROMs are considered the gold standard for measuring outcomes of subjective experiences because the information gained directly from patients reflects their main concerns and problems [8]. However, implementing outcome measures in clinical practice is complex and needs to be adapted to the unique setting, especially in palliative care, as recommended by the European Association for Palliative Care (EAPC) [13, 14]. The PROMs most frequently used in palliative care are the Edmonton Symptom Assessment System (ESAS) [15], which measures perceived symptom severity, and the Integrated Palliative Care Outcome Scale (IPOS) [15, 16], which provides multidimensional perspectives on a patient’s situation, including physical, psychological, social, emotional, and spiritual concerns and needs [17]. Implementation of PROMs can be facilitated with the use of questionnaires, which are considered useful, valid and relevant for the population of interest, and the training and support that organizations provide to HCPs [9, 13, 18]. In palliative care, the use of PROMs allows timely monitoring and management of the symptoms that most affect daily life for terminally ill patients [2, 19, 20]. There is also evidence that PROMs and their systematic use have improved the identification of unmet patient needs and enhanced a larger number of actions based on quality-of-life data [2, 9, 21–26].

Usually, PROMs are compiled in paper format; however, the growth of electronic health (eHealth) technologies and the effects of the COVID-19 pandemic have prompted the collection of electronic PROMs (e-PROMs) using digital networks or devices, such as touchscreen tablets, smartphones, and computers [27–29].

Some advantages of e-PROMs are the accuracy and efficiency of data, the reduction in data entry errors, and the quick availability of the recorded symptom data when HCPs meet the patient [22, 27, 30–33]. However, the implementation of e-PROMs in oncology palliative care may be influenced by various factors, such as cultural and socioeconomic factors, as well as e-health literacy, care setting (inpatient vs. outpatient) and worsening functional health status [2].

HCPs stated that they prefer the use of PROMs in electronic format [34], even though they perceive several barriers in the adoption of e-PROMS in daily practice, such as limited time, the necessity of training, the unfamiliarity of many patients with electronic devices and an inability for patients to complete e-PROMS independently [5, 35–38]. Understanding barriers and facilitators is fundamental to successfully implementing PROMs; different conceptual frameworks have been used to identify barriers and facilitators and to determine the factors that influence implementation [18, 38–40].

A better understanding of health-care professionals’ perspectives on facilitators and barriers to implementing a standardized e-PROMs collection system in clinical practice, is helpful to better implement them in clinical care [39].

In recent decades, more patients in need of palliative care have been cared for in their homes, which increases satisfaction and quality of life in patients and their families [1, 15].

However, few studies have explored HCPs’ perceptions of using PROMs in the home palliative cancer care context. E-PROMs implementations in this care setting need to ensure the acceptability of HCPs and patients and assess their barriers to implementation [24]. HCPs’ views are required to shape how e-PROMs could be embedded within clinical practice [41].

### Aim

The present study explores barriers and facilitators to the adoption of a standardized e-PROM collection system perceived by HCPs (physicians, nurses) in home palliative cancer care.

## Methods

### Design

A qualitative descriptive study design using focus groups was chosen to comprehensively describe the research phenomenon [42]. We followed the Consolidated Criteria for Reporting Qualitative Research (COREQ) [43] to ensure transparency and improve rigor. The current study is part of a larger ongoing project, “Impact assessment of a system e-Patient Reported Outcome Measures on home palliative care: Mixed-methods study of feasibility and intervention”, which aims to promote the use of e-PROMs in palliative cancer care at home in Fondazione IRCCS Istituto Nazionale dei Tumori of Milan (INT).

INT is a comprehensive cancer center in Italy associated with the Organization of European Cancer Institutes, pursuing the prevention, early diagnosis, and treatment of cancer, acknowledging its exceptional standards in patient care and advancement of novel treatments. It specializes in clinical and translational research in the fields of biomedicine and public health, with the primary goal of enhancing the quality of healthcare services.

### Participants

The study took place within the Complex Unit of Palliative Care - Hospice, Pain Therapy, and Rehabilitation at INT. Eligibility for participation in the study was limited to physicians and nurses directly involved in providing home palliative care to patients. Currently, the home care team comprises five physicians and five nurses. Recruitment was undertaken by convenience and purposive sampling. The objectives and methods of the study were explained to all participants. They had the opportunity to ask questions before signing the consent forms and being assigned to a focus group.

HCPs were inexperienced with ePROMs in clinical practice, but they received a prior training program from researchers on how to use them and which PROMs are most frequently used in palliative care.

### Data collection

We explored HCPs’ desired characteristics for e-PROMs regarding future implementation in home palliative cancer care. Data were collected from semistructured focus groups and field notes always on the same sample recruited. Field notes were collected by two observers, IB in the first focus group and DR in the second focus group. According to the literature, ten participants were enough to conduct a focus group [44]. After a first focus group, a second was carried out to examine more deeply the data that emerged in the first focus group.

Data saturation, defined as the point in data collection and analysis with no new information produced, was reached in the second focus group. According to Guest et al., within two to three focus groups, over 80% of the themes can be identified [45].

The guiding questions for the focus groups were structured according to the conceptual framework of Grol et al. concerning the implementation of change in clinical practice, as used in the study by Graupner et al. [38, 46] We collected data on potential barriers and facilitators of the implementation of e-PROMs and assigned findings as one of six themes of Grol’s framework: Innovation (e.g., advantages, advantages in practice, feasibility, credibility, accessibility, attractiveness); Individual professional (e.g., awareness, knowledge, attitude, motivation to change, behavioral routines); Patient (e.g., knowledge, skills, attitude, compliance); Social context (e.g., opinion of colleagues, collaboration); Organizational context (e.g., organization of care processes, staff, capacities, resources, structures); Economic and political context (e.g., financial arrangements, regulations, policies). The researchers who developed the interview guide have backgrounds in palliative care (AC, TC, IB), cancer care (SC, DR), and qualitative research (LC, ML). In Supplementary Material 1 is reported the interview guide of the two focus groups realized by consulting the literature. The guiding questions were modified during the process; before the second focus group, items were added as new relevant themes emerged during the first focus group. Both focus groups were conducted by the first author (LC), with a moderator (SC) and an observer (IB, DR). The focus group meetings took place in a meeting room in the palliative care unit. Paper copies of PROMS (e.g., ESAS or IPOS), were provided in advance to both groups to facilitate discussion, as HCPs had no experience in their use. We asked them to think about it on a tablet because PROMs in electronic format are not yet available at INT at that moment. The focus group met between September and October 2022. The first and second focus group meetings lasted 90 and 30 min, respectively.

### Data analysis

Audio recordings of all focus groups were transcribed verbatim and managed using NVivo V1.6.2 [47]. Grol’s framework was used as a systematic approach to deductively analyze data regarding the factors related to barriers and facilitators of the use of e-PROMs in palliative cancer care at home. Data analyses started directly after the first focus group using direct “Reflexive Thematic Analysis” [48, 49].

Qualitative data were coded line by line independently by LC and IB for the first focus group and by LC and DR for the second focus group. The codes were then discussed by the entire research team. Agreement on the coding was reached during consensus meetings with the senior researcher (ML), an expert in qualitative research. Focus groups transcripts were read critically by researchers; all codes that emerged were grouped into subthemes according to Grol’s framework and then facilitators or barriers were designated. The codes and subthemes from the first and second focus groups were compared, and similar codes and subthemes were grouped together.

### Ethical considerations

Participants provided written informed consent. They were also assured of anonymity and confidentiality of collected data and audio files. Data protection procedures were observed; the data generated and analyzed during the study were stored and protected in a secure location and are therefore not available to the public. Ethical approval was obtained from the Fondazione IRCCS Istituto Nazionale dei Tumori of Milan (INT) Ethics Committee [Ref: 187/21].

Data collection and management were performed in accordance with the tenets of the 1964 Helsinki Declaration and its later amendments and with Italian regulations.

### Trustworthiness

To ensure trustworthiness [50, 51], we applied the following strategies: data triangulation (i.e., researchers analyzed the same data independently), member checking (i.e., a return of the complete focus group analysis to the participants) and peer debriefing (i.e., meetings between researchers were scheduled to allow transcripts, codes and subthemes to be reviewed and evaluated together).

## Results

The baseline characteristics of the HCPs are presented in Table 1; nine HCPs attended the first focus group, and ten attended the second.

**Table 1:** Characteristics of focus group participants

	Number of participants N (%) in Focus Group 1	Number of participants N(%) in Focus group 2
**Sex**		
Male	5 (55)	6 (60)
Female	4 (44)	4 (40)
Age		
< 30	1 (11)	ND
31–40	2 (22)	4 (40)
41–50	3 (33)	3 (30)
51–60	1 (11)	ND
> 61	2 (22)	3 (30)
Type of healthcare professional		
Nurse	5 (55)	5 (50)
Physician	4 (44)	5 (50)
Numbers of Years in Practice		
1–10	2 (22)	2 (20)
11–20	4 (44)	5 (50)
21–30	3 (33)	3 (30)
Numbers of Years in Palliative Care		
1–10	3 (33)	5 (50)
11–20	5 (55)	5 (50)
21–30	1 (11)	ND

*Legend*: ND, no date

**Fig. 1:**
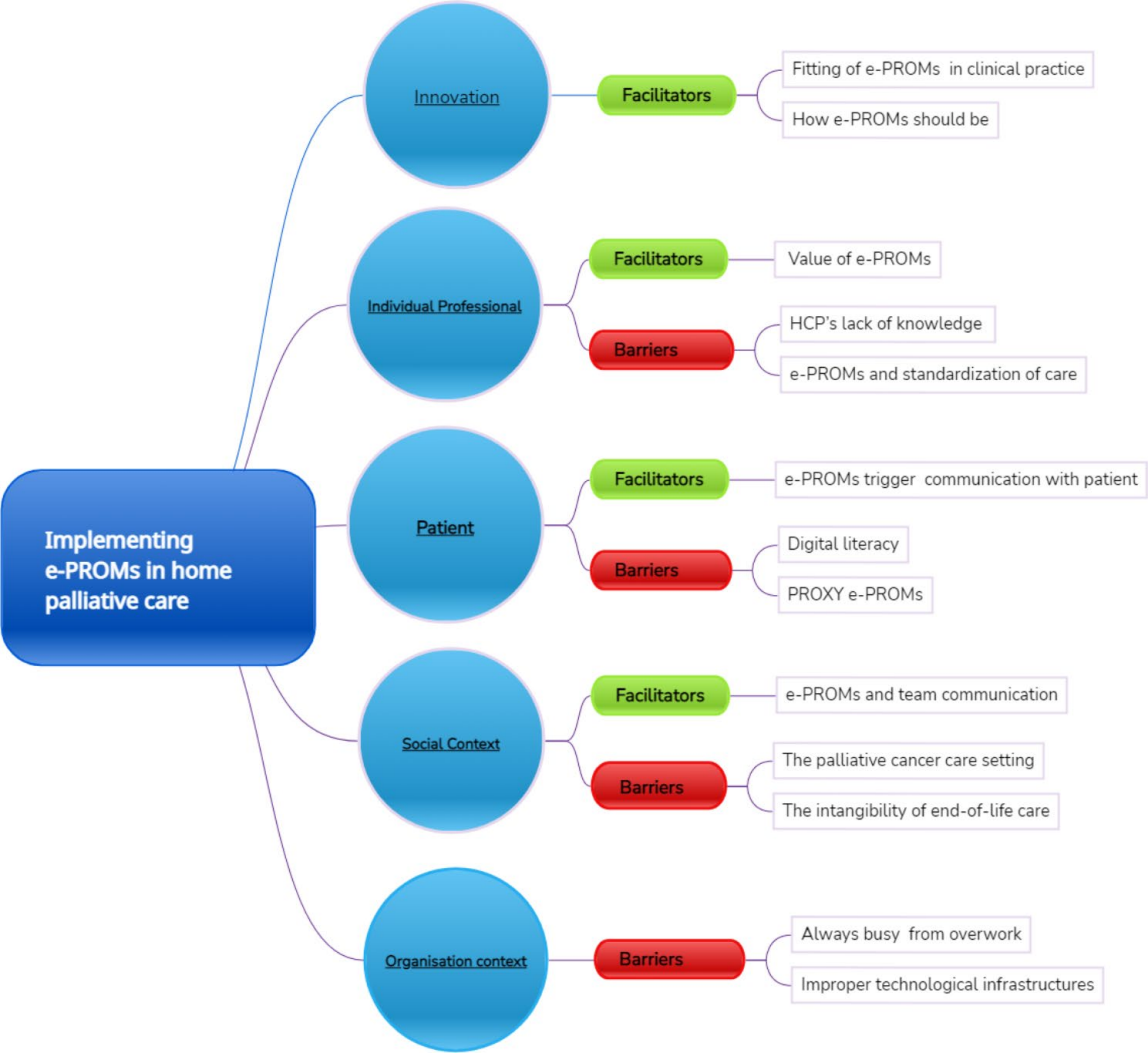
Conceptual map of themes and subthemes according to Grol’s Framework

After analysis, the data were placed in two main categories: barriers and facilitators. A total of 245 codes were identified in the coding process (171 for the first focus group and 74 for the second) [46]. We categorized the identified barriers and facilitators into five of six Grol’s themes. No barriers or facilitators were found in the economic context and regulations theme. Figure 1 represents the conceptual map of themes and subthemes. In the five themes, we identified 13 subthemes containing factors that can be barriers or facilitators; for example, the HCPs agreed that the e-PROMs can standardize clinical visits. However, they are concerned that the e-PROMs could negatively impact patient relationships. The results are summarized in Tables 2 and 3.

**Table 2:** Facilitators of the implementation of e-PROMs

Grol’s Framework Themes	Subthemes	Quotes from focus groups
Innovation	Fitting of e-PROMs in clinical practice	“There are lots of things that patients do not tell you, this would be a way to ask for all the information you then need to decide…” *Physician C* “The key thing for it to work, like any tool, is to be able to do it in front, at the moment in front of the patient.“ *Nurse D*
	How e-PROMs should be	“Things that are included are usable and useful for my work.“ Nurse S “An e-PROMS, easy to have filled out.” *Physician D* “I would need it in such a way that in everyday life I don’t forget to investigate all the points… This would standardize the care provided, because we ask all patients the same things. Therefore, we go and investigate the same points.“ *Nurse S*
Individual professional	Value of e-PRO“S	“If the aim is to improve the care aspect, they are certainly useful.“ *Nurse E* “It is useful because it gathers all the information you need.“ *Physician P* “However, if you look over time in the medical record, you see that the patient did not have dyspnea. Then, the dyspnea got worse. It got worse. Okay, so today it did not appear. I do not send him to the emergency room, I sedate him. The utility maybe could be something like this: pain, pain, pain. I decide to increase the patch. I increase, I increase, I do not increase the patch. I change therapy completely. The utility maybe is this, among us.“ *Physician P*
Patient	e-PROMs trigger communication with patient	“Useful because it is a starting point for the examination”. *Physician P* “However, you see all the symptoms, you see the patient’s problem, you start from there”. *Physician P* “It could be a guide, in the sense that I always ask the same thing at each visit.“ *Nurse S*
Social context	e-PROMS and team communication	“While the core information and the confrontation between professionals, etc., my philosophy of thinking is that at the base there has to be a multidisciplinary approach.“ *Nurse G* “e-PROMS could help compensate for the lack of communication.“ *Physician P*

**Table 3:** Barriers to the implementation of e-PROMs

Grol’s Framework Themes	Subthemes	Quotes from Focus Groups
Patient	“Digital literacy”	“e-PROMs are not a burden on the patient.” *Physician P* “First, the average person in Italy is not at all digitized.“ *Physician C* “They are old people who are not… familiar with these technological things anyway.“ *Physician C*
	“PROXY e-PROMS”	“I am worried. It is not about outpatients, who in theory should be a little better, but very complicated home patients, who are sometimes very sick.” *Nurse G* “Many will not be able to fill out the e-PROMs.“ *Nurse S* “Even patients who are younger, that you can have a dialog with… but there are patients who are in the very late stage of the disease or who are of a certain age that if I go and give them one more thing, it becomes a bit more difficult for them but also for us. I do not know.“ *Nurse G* “It all depends on one patient to another because each patient has his own problems and difficulties and so you also have to account for that many times it might not be self-filled in… but be filled in by the family member.“ *Physician C* “However, in the end when the patient does not make it… you have to work on the perception of the relative…” *Nurse S* “The problem is that the perception of the relative is different from that of the patient.“ *Physician P*
Individual professional	HPC’s lack of knowledge	“Mentality is difficult to change after years and years of various categories of generations.“ *Nurse G* “Because if it is a PROMS that gets filed and afterward I do not even see it, who cares that I filled out a PROMS? It becomes like the… what’s it called…. the satisfaction questionnaire that gets filed and nobody looks at it, so much so that nobody fills it out.“ *Physician D* “We have no experience, so… I do not know if they can be used by us!“ *Physician C* “If we see it as an additional burden you do not need the PROMS to be filled in every time”. *Physician C* “The filling in during the visit is very difficult.“ *Physician P* “In my opinion for the purposes of quality of care, it matters much more the direct relationship of the problems to be addressed with the professionals, more than… the IPOS. I see it more as something that could be useful but more for the purposes of standardization, as it was said before, of education, which are the various areas to be investigated; but to use it as a tool, as a facilitator…” *Physician M* “I’m not saying the timing, the timing. The risk I see is that it becomes just a routine thing anyway, done just to do it.“ *Nurse D* “In my opinion, we need to remove bureaucracy and not increase it.“ *Nurse G*
	e-PROMS and standardization of care	“All this stuff here, which maybe could… which is the heart of our assistance, could come a little bit less.“ Nurse S “It might get in the way because all the things we do, all the soft skills we put in… the field might not be as good.“ *Nurse I* “It is complicated to follow a chart, the visit is so subjective, so articulate and natural.“ *Physician C*
Social context	The palliative cancer care setting	“If you propose this to them on the ward and in the hospital, and make them do it, I think they will fill in or self-complete because they feel a little bit in awe anyway.“ *Physician P* “I do not know if home care is a suitable setting for this type of PROMs.“ *Physician P* “When we go home we must always remember that we are guests.“ *Physician C*
	The intangibility of end-of-life care	“What can we do truly effectively to improve the condition.“ *Nurse G* “However, objectively how many times do we do it? So many words of support and that is it, and our presence is not an intervention. You do not do anything clinically, but… the patient is happy like that.“ *Nurse S* “The beauty of our care is that there are no limits. There are no boundaries, and what we do is what the patient wants to do at that moment.“ *Nurse G*
Organizational context	Always busy from overwork	“Already filled out this form, start a quarter of an hour at the first visit, ten minutes, five minutes at the end. Therefore, I have to do a lot of thing, and e-PROMs could take too much time.” *Nurse S* “I am afraid that the e-PROMs may take up my time during the visit.” *Nurse I*
	Improper technological infrastructures	“We have very slow computer systems.” *Physician C* “We cannot objectively enter everything in real time, at home to access the file takes a long time, so you do not use it”. *Nurse S* “I would need PROMs reports ready immediately, easy to understand and visible right away” *Nurse S*

### Facilitators

#### Innovation

*Subthemes: “*Fitting of e-PROMs in clinical practice” and “How e-PROMs should be”.

This theme brings out only facilitators perceived, despite neither physicians nor nurses having used e-PROMs in practice; the HCPs said they think that implementing e-PROMs can help patients systematically highlight perceptions or concerns about their health status. In standard care, these aspects may not be reported by patients. The HCPs agreed that the PROMs must reflect the end-of-life needs of oncological patients being cared for at home.

Moreover, the HCPs stressed the need for patients to play an integral part in the choice of the PROMS, which must have well-defined characteristics in terms of practicality and usability. HCPs stated that they would like to incorporate these tools in their practice to avoid further aggravating in a complex setting such as palliative oncological home care.

#### Individual professional

##### Subtheme

“Value of e-PROMs”.

HCPs perceive the possible integration of e-PROMS as beneficial for their clinical activity at home. e-PROMs can provide a more effective response to the patient’s needs and provide a complete overall picture of the patient’s health status.

The objective is to improve the care provided at home by having the patient assess his or her symptoms so that HCPs can respond with better control and management of symptoms over time that the patient experiences. According to HCPs, e-PROMs could guide clinical choices related to symptom treatment to better direct practice.

#### Patient

##### Subtheme

“e-PROMs trigger communication with patient”.

HCPs perceive the use of e-PROMS as an excellent starting point for communication with the patient during the home visit. The ability for the patient to state at the beginning of the stay how they are feeling or what is bothering them makes it possible to improve the relationship between the patient and HCP, even for patients who are less willing to relate to HCPs.

#### Social context

##### Subtheme

“e-PROMS and team communication”.

In this context of home palliative cancer care, physicians and nurses make home visits separately; this sometimes makes the passing of information fragmented, making all of the pieces available only at weekly team meetings. The focus groups show that integrating e-PROMs into the medical record could improve simultaneous consultation of patient-reported health status by all HCPs and, consequently, critical shared decision-making; they would also enable reflection in plenary with the entire staff during weekly meetings.

### Barriers

#### Individual professional

*Subthemes: “*HCP’s lack of knowledge” and “e-PROMs and standardization of care”.

HCPs expressed reluctance to use the e-PROMs because they had never used this kind of questionnaire before. The implementation requires a significant change in their work and a possible activity slowdown. Staff also think that the process is difficult due to increased workload.

A barrier reported by HCPs is the rigidity of e-PROMs, which might limit the personalization of home visits, reducing the spontaneity of the care relationship.

#### Patient

##### Subthemes

“Digital literacy” and “PROXY *e–PROMs”*.

All HCPs expressed concern about the completion of e-PROMs by the patients in home care. The users of palliative oncological home care are often elderly persons who are unfamiliar with technological tools or people in poor general condition due to advanced oncological disease. Sometimes, due to sedation or incoercible symptoms, they are not able to self-complete the e-PROMs. In these cases, the caregiver becomes the one who materially fills out the electronic questionnaire as if they were the patient themselves. However, the HCPs know that the caregiver often has a different view than the patient.

#### Social context

*Subthemes* “The palliative cancer care setting” and “The intangibility of end-of-life care”.

HCPs underline that the care setting is the patient’s home and emphasize respecting the personal and private context. HCPs said that sometimes they felt like guests in the patient’s homes. In this setting, it is often impossible to plan clinical schedules and activities because assessing the patient’s needs at that time is essential. HCPs report that often no tangible and material interventions are necessary for patients, that they just need support and closeness.

#### Organizational context

##### Subthemes

“Always busy from overwork” and “Improper technological infrastructures”.

In this theme, there are only barriers. HCPs state that home visits are often very time-consuming, and the compilation of e-PROMs could increase their time workloads. Furthermore, an issue strongly emphasized by all team members is the inadequacy of the computer systems in use. Although PROMs are designed to facilitate care, available technology slows it down and complicates it, creating dissatisfaction due to a lack of practicality.

## Discussion

This study aimed to detect barriers and facilitators perceived by palliative physicians and nurses who work at home to implementing e-PROMs in a complex setting such as at-home palliative cancer care. The enrolled HCPs had no experience but only training with e-PROMs; for their implementation, it was necessary to understand the HCPs’ point of view, according to Amini et al. [39]. The barriers and facilitators that emerged are congruent with previous studies.

According to the literature, e-PROMs could be integrated into clinical practice after adequate training and support of HCPs at all stages of implementation [13, 35], including the choice of the most suitable tool and the education of patients in their use.

In their systematic review, Antunes et al. identified staff motivation in adopting PROMS in palliative care, influenced by their perception of how these tools can enhance and help them in clinical practice [8]; this also reflects our findings. Indeed, HCPs see an added value in e-PROMs for their practice and would be willing to use these tools since they consider them useful and valid, especially for clinical decision-making [8, 9, 20, 25, 26]. Implementation and development of e-PROMs should be geared toward capturing the salient aspects of the health status of these patients, enabling better control of their symptoms. e-PROMs may represent an input of collective and shared reflection, triggering discussion during the clinical visit with the patient at home [2, 5] and improving more open and deep communication between patients and the entire healthcare team [15, 21, 38].

Furthermore, they should be easy to complete in terms of the length of questionnaires and the speed of technological systems. As reported by Graupner et al. [38], technological solutions for e-PROMs support may be considered facilitators of patient care if they are efficient and fast or barriers if they are slow and underperforming. Indeed, our HCPs thought that e-PROMs could increase their working time and consequently their workload [13, 21].

The uniqueness of the palliative care context brings out another barrier linked to the worry that e-PROMs could standardize home visits to the detriment of the naturalness that these patients need to maintain in those moments. Proximity, listening and intangible care interventions are often needed, but HCPs fear that standardization might make them feel the risk of low humanization of care. The end of life and patients’ homes represent two key concepts to be considered; the patient’s home is a special care setting, different from the hospital. Patients welcome HCPs into their homes, to a place that belongs to them and they know well, where they feel protected. Visits can sometimes interfere with their daily routines, which should be maintained and respected. Innovations, especially things unknown and far from normal, may appear as a threat; in fact, patients are often frail elderly and unfamiliar with technology [33].

Furthermore, the condition of terminality often results in severe physical/mental deterioration that makes it impossible for patients to compile e-PROMs [8, 9, 15], which are left to the caregivers; even if proxy-PROMs provide useful data for HCPs, these data may be distorted by the experience and perception of the caregivers [26].

In agreement with other studies, more barriers than facilitators for e-PROMS were evinced in focus groups [5, 18], which were related to professionals’ lack of knowledge about e-PROMs. This reflects their skepticism regarding the applicability of such systems within the context in which they work [18, 21]. In implementing these tools, the uniqueness of the care setting and the end-of-life patients must be kept in mind, respecting the delicacy of the moment these people and their families are facing.

### Strengths and limitations

One of the strengths of this study is that it is part of a bigger project with mixed method.

Furthermore, the methodology used in this study is well suited to address the research questions. The focus groups explored perceptions of home palliative cancer care HCPs and gained new data on the possible barriers to the implementation of e-PROMs in this setting. Data collection and analysis by multiple researchers enabled extensive involvement with the data and understanding of context. This study has some limitations due to the nature of qualitative research and single-center sampling; this limits the generalizability of the results.

## Conclusions

The implementation of e-PROMs in home palliative cancer care entails a change in clinical practice that needs to be carefully integrated. E-PROMs are perceived by HCPs as adding value to patient care and their work; however, barriers remain especially related to the fragility of this population, the adequacy of technological systems, lack of education, and the risk of low humanization of care. Understanding the barriers perceived by HCPs is crucial to successfully implementing these tools in clinical practice. Future research should also analyze palliative cancer patients’ perceptions of the use of e-PROMs in their care at home.

### Electronic supplementary material

Below is the link to the electronic supplementary material.

Supplementary Material 1

## Data Availability

The datasets generated and analyzed during the current study are not publicly available due to a potential lack of anonymity within the full focus groups. ***Competing interests***. The authors declare that they have no competing interests.

## List of abbreviations

PC: Palliative care
PROs: Patient-reported outcomes
eHealth: electronic health
HCPs: Healthcare professionals
PROMs: Patient-reported outcome measures
EAPC: European Association for Palliative Care
ESAS: Edmonton Symptom Assessment System
IPOS: Integrated Palliative Care Outcome Scale
e-PROMs: Electronic patient-reported outcome measures
INT: Fondazione IRCCS Istituto Nazionale dei Tumori of Milan
Research COREQ: Consolidated Criteria for Reporting Qualitative Research
