# Supplementary material for: Barriers and facilitators of electronic patient-reported outcome measures (e-PROMs) for patients in home palliative cancer care: a qualitative study of healthcare professionals’ perceptions
Source: BMC Palliat Care. 2023 Aug 4;22:111. doi: 10.1186/s12904-023-01234-0 (PMC10401773; doi:10.1186/s12904-023-01234-0)
Supplement: Supplementary file 1 — Supplementary Material 1 [file 12904_2023_1234_MOESM1_ESM.docx]

| Interview Guide |
| --- |
| 1. What are your views on the collection and use of e-PROMs in your clinical practice?    1. Probe: Individual professional (e.g. knowledge, attitude, motivation to change, behavioral routines)    2. Probe: Patient (e.g., knowledge, skills, attitude, compliance)       1. Prompts: benefits, barriers 2. How do you think the routine collection of these data can be used in your clinical practice?    1. Probe: Innovation (e.g., advantages, advantages in practice, credibility, accessibility, attractiveness)    2. Probe: Organizational context (e.g., organization of care processes, staff, capacities, resources, structures)       1. Prompts: feasibility, facilitating use, training 3. The literature suggests that collecting this data and providing it to healthcare professionals may affect patient care. How do you think this will happen in practice?    1. Probe: Innovation (e.g., advantages, advantages in practice, feasibility)    2. Probe: Organizational context (e.g., organization of care processes, staff, capacities, resources, structures)       1. Prompts: changes in practice 4. How would you like the e-PROMs questionnaire to be?    1. Probe: Individual professional (e.g., attitude, motivation to change, behavioral routines)    2. Probe: Social context (e.g., collaboration)    3. Probe: Organizational context (e.g., organization of care processes, staff, capacities)       1. Prompts: feasibility, facilitating use, training 5. Do you think it would be more useful to fill out e-PROMs during the home visit with the patient next to you? Or have patients use them when necessary to promptly report their health status?    1. Probe: Social context (e.g., collaboration)    2. Probe: Organizational context (e.g., organization of care processes, staff, capacities)       1. Prompts: organization of care processes, staff, capacities 6. Would you have them filled out at each visit or at set intervals?    1. Probe: Social context (e.g., collaboration)    2. Probe: Individual professional (e.g., attitude, motivation to change, behavioral routines)    3. Probe: Patient (e.g., attitude, compliance)       1. Prompts: organization of care processes, staff, capacities 7. In your opinion, which factors could be an obstacle to administering e-PROMs to patients?    1. Probe: Patient (e.g., knowledge, skills, attitude, compliance)    2. Probe: Economic and political context (e.g., financial arrangements)       1. Prompts: skills, attitude, compliance, policies and regulations |

**Additional File 1: Interview Guide**
